# Differences between men and women in dietary intakes and metabolic profile in response to a 12-week nutritional intervention promoting the Mediterranean diet

**DOI:** 10.1017/jns.2015.2

**Published:** 2015-04-13

**Authors:** Vicky Leblanc, Anne-Marie Hudon, Marie-Michelle Royer, Louise Corneau, Sylvie Dodin, Catherine Bégin, Simone Lemieux

**Affiliations:** 1 Institute of Nutrition and Functional Foods, Laval University, 2440 Hochelaga Boulevard, Québec, Canada, G1V 0A6; 2 Department of Obstetric and Gynaecology, Laval University, Pavillon Ferdinand-Vandry, 1050 Medicine Avenue, Québec, Canada, G1V 0A6; 3 School of Psychology, Laval University, Pavillon Félix-Antoine Savard, 2325 rue des Bibliothèques, Québec, Canada, G1V 0A6

**Keywords:** Differences between men and women, Mediterranean diet, Cardiovascular risk, Self-determination theory, GLM, general linear model, HDL-C, HDL-cholesterol, Medscore, Mediterranean score, LDL-C, LDL-cholesterol, SDT, self-determination theory, total-C, total cholesterol

## Abstract

Few studies have compared men and women in response to nutritional interventions but none has assessed differences between men and women in the response to a nutritional intervention programme based on the self-determination theory (SDT) and using the Mediterranean diet (MedDiet) as a model of healthy eating, in a context of CVD prevention and within a non-Mediterranean population. The present study aimed to document differences between men and women in changes in dietary, anthropometric and metabolic variables, in response to a nutritional intervention programme promoting the adoption of the MedDiet and based on the SDT. A total of sixty-four men and fifty-nine premenopausal women presenting risk factors for CVD were recruited through different media advertisements in the Québec City Metropolitan area (Canada). The 12-week nutritional programme used a motivational interviewing approach and included individual and group sessions. A validated FFQ was administered to evaluate dietary intakes from which a Mediterranean score (Medscore) was derived. Both men and women significantly increased their Medscore in response to the intervention (*P* < 0·0001). Men showed a significantly greater decrease in red and processed meat (−0·4 (95 % CI −0·7, −0·1) portions per d) and a greater increase in fruit (0·9 (95 % CI 0·2, 1·6) portions per d) intakes than women. Significant decreases were observed for BMI and waist circumference in both men and women (*P* ≤ 0·04). Significant greater decreases were found for total cholesterol (total-C):HDL-cholesterol (HDL-C) (−0·2; 95 % CI −0·4, −0·03) and TAG:HDL-C (−0·2; 95 % CI −0·4, −0·04) ratios in men than in women. When adjusting for the baseline value of the response variable, differences between men and women became non-significant for red and processed meat and fruit intakes whereas significant differences between men and women (i.e. larger increases in men than women) were observed for legumes, nuts and seeds (0·6 (95 % CI 0·2, 1·0) portions per d) and whole-grain products (0·5 (95 % CI 0·01, 1·0) portions per d) intakes. For metabolic variables, differences between men and women became non-significant for total-C:HDL-C and TAG:HDL-C ratios when adjusted for the baseline value of the response variable. The present results suggest that the nutritional intervention promoting the adoption of the Mediterranean diet and based on the SDT led to greater improvements in dietary intakes in men than in women, which appear to have contributed to beneficial anthropometric and metabolic changes, more particularly in men. However, the more deteriorated metabolic profile found in men at baseline seems to contribute to a large extent to the more beneficial changes in CVD risk factors observed in men as compared with women.

Adoption of healthy eating habits is encouraged in the context of chronic disease prevention and the Mediterranean diet has been ranked as one of the best models to provide protection against CVD^(^^1^^–^^3^^)^. The Mediterranean diet pattern is characterised by a high intake of vegetables, fruits, legumes, nuts, cereals (mainly unrefined), a high intake of olive oil, a low-to-moderate intake of dairy products, a low intake of meat and poultry, and a regular but moderate intake of alcohol, primarily in the form of wine and generally during meals^(^^3^^)^.

Studying differences between men and women in response to interventions aimed at preventing or treating diseases is absolutely essential for providing optimal care to men and women. Without such studies comparing men and women, we would not know, for example, that usage of some medications for preventing or treating CVD are efficacious in men but not appropriate in women^(^^4^^,^^5^^)^. At this point it is essential to underline that differences observed between men and women can be explained by both sex and gender differences. Sex differences refer to biological and physiological characteristics that distinguish males from females while gender is described as socially constructed roles, relationships, behaviours, relative to power, and other traits that societies ascribe to men and women^(^^6^^)^. When studying differences between men and women in response to nutritional interventions, both sex and gender differences can be involved to a different degree depending upon the type of intervention and the two constructs have been suggested to be closely interrelated and difficult to dissociate^(^^6^^)^.

Some studies have documented differences between men and women in the context of controlled studies where all food and drinks are provided. In such a context, differences observed between men and women in response to the intervention refer more to sex than to gender differences^(^^7^^–^^9^^)^. In fact, in those types of studies the impact of the diet on metabolic variables measured can be influenced by sex-related factors such as sex hormones^(^^10^^)^ and is not likely to be influenced by factors such as diet adherence that is in turn modulated by gender-related factors. A few studies have been performed to compare men and women in response to diet manipulations performed in controlled conditions. Accordingly, a greater decrease has been reported in LDL-cholesterol (LDL-C) levels in response to a low-SFA diet in men than in women^(^^8^^,^^9^^,^^11^^)^ and a recent study published by our team showed decreases in insulin levels in men but not in women, in response to a 4-week Mediterranean diet^(^^7^^)^. On the other hand, in nutritional interventions during which subjects continue to buy their food, cook their meals and make decisions about what they eat, the differences observed between men and women cannot be considered as sex differences since gender-specific factors such as attitudes, beliefs and motivation towards food regulation are influencing adherence to dietary recommendations and therefore health benefits that can be obtained from it. This is why when referring to these types of studies the term gender differences is more appropriate.

Only a few studies have been performed to assess gender differences in response to educational nutrition programme promoting the Mediterranean diet. Among a Mediterranean population, a higher success in improving adherence to the Mediterranean diet in men than women was reported after 1 year in the PREDIMED trial, which includes Spanish men and women presenting high risk for CVD^(^^12^^)^. Among a non-Mediterranean population, a study has reported the impact of a Mediterranean diet education programme in hypercholesterolaemic men and women and showed that whereas women improved their dietary intakes in accordance with the education programme and significantly decreased their total cholesterol (total-C) levels, no such changes in serum total-C were observed in men^(^^13^^)^.

Changing eating habits represents a major challenge for many individuals^(^^14^^)^ and evidence indicates that the extent to which health professionals involve their clients in the decision-making process may influence adherence to treatment^(^^15^^)^. In this regard, the self-determination theory (SDT) suggests that stimulating optimal quality of motivation could help individuals to evolve toward healthier eating habits^(^^16^^)^. To the best of our knowledge, no study has assessed differences between men and women in the response to a nutritional intervention programme based on the SDT and using the Mediterranean diet as a model of healthy eating, in a context of CVD prevention and within a non-Mediterranean population. Therefore, the objective of the present study was to determine differences between men and women in changes in dietary, anthropometric and metabolic variables, in response to a 12-week nutritional intervention programme promoting the adoption of the Mediterranean diet, based on the SDT, in Canadian men and women presenting risk factors for CVD.

## Methods

### Participants

The present study was conducted among a sample of sixty-four men and fifty-nine premenopausal women aged between 25 and 50 years, and were recruited through different media advertisements in the Québec City Metropolitan area (Canada). In women, a follicle-stimulating hormone (FSH) measurement was performed if needed (for example, when women presented menstrual irregularities) to confirm premenopausal status (FSH < 20 IU/l)^(^^17^^)^. Men and women had to present slightly elevated LDL-C concentrations (between 3·0 and 4·9 mmol/l)^(^^18^^)^ or a total-C:HDL-cholesterol (HDL-C) ratio ≥ 5·0, and at least one of the four following criteria of the metabolic syndrome^(^^19^^)^: (1) TAG concentrations ≥ 1.7 mmol/l; (2) fasting glycaemia between 6·1 and 6·9 mmol/l; (3) blood pressure measurements ≥ 130/85 mmHg; and (4) waist circumference ≥ 80 cm in women and ≥ 94 cm in men^(^^20^^)^. Participants also had to have a stable body weight (± 2·5 kg) for a minimum of 3 months before the beginning of the study and to be involved in food purchases and/or preparation at home. We excluded men and women who had cardiovascular events and who used medication that could affect dependent variables under study, i.e. medication for hypertension, dyslipidaemia and diabetes (type 1 and type 2). Pregnant women, smokers, participants with an alcoholism history or with a high Mediterranean score (Medscore > 29; i.e. food pattern already highly concordant with the Mediterranean diet)^(^^21^^)^ were also excluded. The present study was conducted according to the guidelines laid down in the Declaration of Helsinki and all procedures involving human subjects were approved by the Laval University Research Ethics Committee on human experimentation. All subjects voluntarily agreed to participate in the research project and written informed consent was obtained from all men and women before their participation in the study. This clinical trial was registered at www.clinicaltrials.gov as NCT01852721.

### Study design

**Fig. 1.**
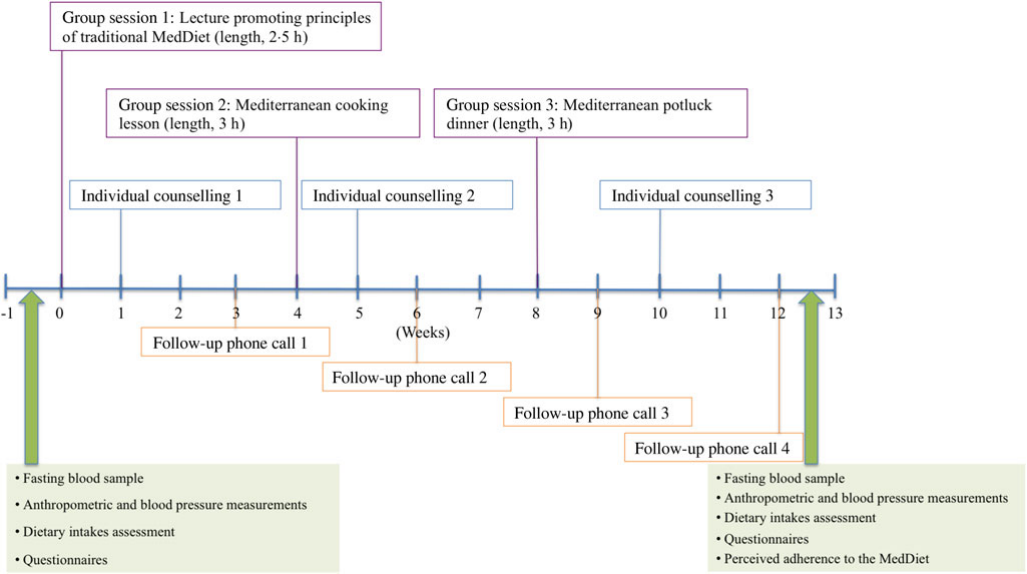
Description of the 12-week nutritional intervention programme and measurements performed at baseline (time = 0) and after the end of the intervention (time = 12 weeks). MedDiet, Mediterranean diet.

The 12-week nutritional programme was based on the SDT and used a motivational interviewing approach. The SDT relies on the quality of the motivation that regulates behaviours, which lies on a continuum from lower to higher self-determined motivation forms (extending from amotivation to intrinsic motivation)^(^^22^^)^. The SDT also postulates that the key component for the development of intrinsic motivation is the satisfaction of basic psychological needs which are autonomy, competence and relatedness^(^^22^^)^. The study was conducted in five phases (spanning from January 2010 to November 2012) and the nutritional intervention included three group sessions (with ten to fifteen individuals each), three individual sessions and four follow-up telephone calls with a registered dietitian (Fig. 1). Three registered dietitians were trained to provide a standardised intervention and participants always met with the same dietitian during individual sessions. The first group session was a lecture, always provided by the same dietitian and aiming at explaining principles of the traditional Mediterranean diet (length: 2·5 h; thirteen to twenty-five participants per group). At week four, men and women actively participated to a 3 h Mediterranean cooking lesson during which they had to cook a Mediterranean meal (eight to fourteen participants per group). At week eight, they shared a 3 h Mediterranean potluck dinner aimed at discussing barriers met in adopting dietary recommendations since the beginning of the intervention (five to twelve participants per group). Individual counselling took place at weeks 1, 5 and 10 and lasted between 45 min and 1 h for each appointment. Individual follow-up telephone calls took place at weeks 3, 6, 9 and 12, and lasted about 20–30 min for each telephone call. The main objective of individual counselling and follow-up telephone calls was to assess dietary changes and to determine progressive personal goals aimed at improving the adherence to Mediterranean diet principles. Different tools such as the decisional balance and the action plan, congruent with the motivational interviewing approach, were used during the individual sessions to formulate dietary objectives while increasing self-determined motivation. In accordance with the SDT^(^^22^^)^, basic psychological needs (i.e. autonomy, competence and relatedness) were promoted during the nutritional intervention via the motivational interviewing approach in order to increase self-determined motivation. More specifically, autonomy and competence of men and women were promoted by the dietitian during individual sessions, i.e. in supporting them into their decision-making process about dietary changes and potential strategies to achieve and maintain these changes, but also during the group sessions by improving their cooking skills and knowledge related to food and nutrition. Therefore, the dietitian had a client-centred approach and put no pressure on participants about the type of dietary objectives to be chosen. In addition, no emphasis was put on body-weight control. Men and women were encouraged to maintain dietary changes in an autonomous way at the end of the nutritional programme and there was no additional contact with the dietitian after the end of the 12-week intervention.

### Measurements of dependent variables

All measurements were performed before (time = 0) and after the 12-week nutritional intervention programme (time = 12 weeks), except for the perceived adherence to the Mediterranean diet which was assessed only at the end of the intervention (time = 12 weeks).

#### Dietary variables

A validated FFQ^(^^23^^)^ was administered by a registered dietitian. The FFQ is based on typical foods available in Québec. It contains ninety-one items and thirty-three subquestions. Participants were questioned about the frequency of intake of different foods and drinks during the last month and could report the frequency of these intakes in terms of day, week or month. A Medscore^(^^21^^)^ was calculated based on the FFQ and allowed to assess the level of adherence to the Mediterranean food pattern. A partial score varying from 0 to 4 is attributed to each of the eleven components of the Mediterranean pyramid. The Medscore could therefore vary between 0 and 44 points. Components of the Medscore are: grains (whole and refined); vegetables (whole and juices); fruits (whole and juices); legumes, nuts and seeds; olive oil (including olives and rapeseed oil); dairy products; fish (including seafoods); poultry; eggs; sweets and red meat/processed meat. As previously described^(^^21^^)^, a high consumption of food groups promoted by the Mediterranean diet (grains, vegetables, fruits, legumes, nuts and seeds, olive oil and fish) contributed to increase the Medscore, whereas a high consumption of food groups less concordant with the Mediterranean diet (sweets and red meat/processed meat) contributed to decrease the Medscore. Moreover, a moderate consumption of dairy products, poultry and eggs obtained the maximum possible score for the respective component. A maximum of one point was respectively attributed to refined grains, vegetables juice, fruit juice consumption and intake of rapeseed oil or margarine made from olive or rapeseed oil. Macronutrient and micronutrient intakes obtained from the FFQ were evaluated using the Nutrition Data System for Research software (NDS-R, version 4.03_31; Nutrition Coordinating Center, University of Minnesota).

#### Anthropometric and metabolic profile

According to standardised procedures^(^^24^^)^ height was measured to the nearest millimetre with a stadiometer (Seca 222 Mechanical Telescopic Stadiometer), body weight was measured to the nearest 0·1 kg on a calibrated balance (BWB-800S Digital scale; Tanita), and BMI was then calculated. Waist circumference measure was also taken to the nearest millimetre according to standardised procedures^(^^24^^)^. Body fat percentage was estimated using the Tanita body-fat analyser, with the accuracy level being ± 5 % of the institutional standard of body composition analysis (dual-energy X-ray absorptiometry) and repeatable to within ± 1 % variation when used under consistent conditions (Tanita-BC-418 body-fat analyser; Tanita Corp.). Blood samples were collected after a 12 h overnight fast. Total-C, HDL-C and TAG concentrations in serum were measured using commercial reagents on a Modular P chemistry analyser (with 0·8 and 1·7 % of within- and between-assay precision, respectively) (Roche Diagnostics). Serum LDL-C concentrations were obtained by calculation using the Friedewald equation^(^^25^^)^ and apoB concentrations by immunoturbidimetry (with < 1·5 and < 2·5 % of within- and between-assay precision, respectively) (Roche Diagnostics). Plasma glucose concentrations were measured with the hexokinase enzymic method (with 0·7 and < 1·2 % of within- and between-assay precision, respectively) and plasma insulin concentrations by electrochemiluminescence (with < 2·0 and < 2·8 % of within- and between-assay precision, respectively) (Roche Diagnostics). Systolic and diastolic blood pressures were measured on the right arm and using an automated blood pressure monitor (BPM 300-BpTRU: Vital Signs Monitor) after a 10 min rest in the sitting position. Measurement of blood pressure was computed as a mean of three readings.

#### Perceived adherence to the Mediterranean diet

At the end of the nutritional intervention (time = 12 weeks), men and women were invited to rate their perception of adherence to the Mediterranean diet principles according to a visual analogue scale (range 0–150 mm). Accordingly, the following question was asked: ‘In your opinion, to what extent do your current dietary intakes meet the Mediterranean diet principles?’ (not at all to perfectly). The distance between 0 mm and the vertical mark drawn on the 150 mm horizontal line was then measured with a ruler and corresponded to the perceived level of adherence to the Mediterranean diet (adapted from Dansinger *et al.*^(^^26^^)^).

### Statistical analyses

Results are first presented in descriptive tables with pre-intervention (time = 0) and post-intervention (time = 12 weeks) mean values (95 % CI) according to men and women (Tables 2 and 4). Then, results are reported as changes within men and within women (Δ values) calculated as post-nutritional intervention minus pre-nutritional intervention values and as percentage of change from baseline value (with *P* value), and two columns with the difference between men and women and the difference between men and women adjusted for the baseline value of the response variable, as mean values and 95 % CI (Tables 3 and 5). Differences between men and women in dietary intakes, anthropometric and metabolic variables were assessed using an ANCOVA (general linear model; GLM procedure) on Δ values. The least squares means (LSMEANS) of the GLM procedure, which can be defined as a linear combination (sum) of the estimated effects, for example, means, from a linear model and based on the model used, allowed determining significant changes in outcomes over time in men and women. The main model of the GLM procedure included gender only, but additional analyses included gender, baseline value of the response variable and gender × baseline value interaction in the model. The interaction was removed from the model when it did not reach statistical significance. Student's *t* test was used to compare macronutrient intakes as well as anthropometric and metabolic variables of men and women before the beginning of the nutritional intervention programme and allowed comparisons of the perceived adherence to the Mediterranean diet between men and women. The χ^2^ test was performed to compare the frequencies of categorical data, i.e. attrition rate and attendance rate to intervention sessions, between men and women. Since three different dietitians were in charge of providing the intervention, the intervener effect was tested using an ANOVA with the GLM procedure. For variables not normally distributed, a transformation was performed but these variables are presented as raw data in the tables. In order to determine sample size, we considered a difference of 35 % in the change in Medscore as being clinically significant, based on results of a previous study from our group^(^^21^^)^. Therefore a final sample size of forty-five men and forty-five women was needed to detect a difference of 35 % in the change in Medscore between men and women with a power of 0·80 and α of 0·05, considering that the standard deviation corresponds to 55 % of the mean of the change in Medscore. The probability level for significance used for the interpretation of all statistical analyses was set at a α level of *P* ≤ 0·05. All analyses were performed using SAS statistical software (version 9.2; SAS Institute Inc.).

## Results

Table 1 shows characteristics of men and women in terms of their age, anthropometric variables and metabolic profile. Men and women included in the present study were about the same age, but men had higher BMI, waist circumference, total-C:HDL-C ratio and TAG levels than women, whereas women had a higher percentage of body fat and HDL-C levels than men.

**Table 1.** Baseline characteristics of men and women (Mean values and standard deviations)

	Men (*n* 64)		Women (*n* 59)	
	Mean	sd	Mean	sd
Age (years)	41·0	7·9	41·8	6·7
BMI (kg/m^2^)	30·8	4·4	29·6*	6·0
Body fat (%)†	26·7	4·5	39·2*	6·2
Waist circumference (cm)	106·1	10·2	95·8*	11·5
LDL-C (mmol/l)‡	3·6	0·7	3·6	0·7
HDL-C (mmol/l)‡	1·1	0·2	1·4*	0·3
Total-C:HDL-C ratio‡	5·1	1·0	4·2*	0·9
TAG (mmol/l)‡	1·9	0·9	1·5*	0·6
Fasting glucose (mmol/l)‡	5·3	0·5	5·2	0·7

LDL-C, LDL-cholesterol; HDL-C, HDL-cholesterol; total-C, total cholesterol.

* Mean value was significantly different from that for men (*P* ≤ 0·05; Student's *t* test).

† Men (*n* 52) and women (*n* 48) because of missing values.

‡ Metabolic variables: men (*n* 63) and women (*n* 58) because of missing values.

Overall, attrition rate was similar in men and women (10·9 and 13·6 %, respectively; *P* = 0·66), and, except for higher LDL-C levels in completers, no significant differences were observed in baseline characteristics of participants who dropped out compared with the ones who completed the 12-week nutritional intervention. Among completers, no differences were observed between men and women for the attendance rate to the whole intervention (8·9 (sd 2·0) sessions in men and 9·0 (sd 1·8) sessions in women, out of a maximum of ten sessions) nor for each component taken separately, i.e. attendance to group meetings (2·3 (sd 0·8) in men and 2·5 (sd 0·7) in women, out of a maximum of three meetings), individual counselling sessions (2·8 (sd 0·6) in men and 2·8 (sd 0·5) in women, out of a maximum of three sessions) and follow-up telephone calls (3·7 (sd 0·9) in men and 3·7 (sd 0·8) in women, out of a maximum of four follow-up telephone calls). Moreover, a significant difference in attendance rate between the different group meetings was observed (*P* ≤ 0·0001), with the higher participation rate found at the lecture on traditional Mediterranean principles (group meeting 1) and the lower rate found at the potluck dinner (group meeting 3). Briefly, 95·2, 82·5 and 57·1 % of men (*P* ≤ 0·0001) and 98·3, 83·1 and 71·2 % of women (*P* ≤ 0·0001) attended group meetings 1, 2 and 3, respectively. Similarly, significant differences in attendance rate between the different individual counselling sessions and also between the different follow-up telephone calls were observed (*P* = 0·002 and *P* = 0·006, respectively), with a progressive decrease over time in the participation rate. The change in Medscore was not influenced by the dietitian in charge of the intervention as indicated by the ANOVA (*F* = 0·36; *P* = 0·70).

Table 2 presents nutritional intakes as well as the Medscore and its components at baseline and at the end of the intervention, and Table 3 presents changes in these variables in response to the 12-week nutritional intervention programme, in men and women separately. Significant differences were found between men and women for changes in energy density, percentage of energy intake provided by lipids, SFA and *trans*-fatty acids and total dietary fibre intake. Indeed, men significantly decreased more their energy density, had a greater increase in total dietary fibre intake, and greater decreases in percentage of energy intake from lipids, SFA and *trans*-fatty acids than women, in response to the intervention. Moreover, both men and women significantly increased the percentage of energy intake provided by PUFA, although no difference was observed between them. Also, a significant decrease in energy intake was observed in men only in response to the intervention. When statistical analyses were adjusted for the baseline value of the response variable, similar results were obtained for the percentage of energy intake provided by SFA and total dietary fibre intake whereas differences between men and women were no longer significant for energy density, percentage of energy intake provided by lipids and *trans*-fatty acids.

**Table 2.** Dietary intakes, Mediterranean score (Medscore) and food group intakes at baseline (time = 0) and after the 12-week nutritional intervention programme (time = 12) (Mean values and 95 % confidence intervals)

	Men				Women			
	Time = 0 (*n* 64)		Time = 12 (*n* 57)		Time = 0 (*n* 59)		Time = 12 (*n* 51)	
Variables	Mean	95 % CI	Mean	95 % CI	Mean	95 % CI	Mean	95 % CI
Dietary intakes								
Energy intake (kJ)	12 824	11 887, 13 761	11 581	10 791, 12 372	10 280*	9657, 10 904	9770	9150, 10 389
Energy density (kJ/g)‡	5·52	5·27, 5·77	5·06	4·81, 5·31	5·06*	4·90, 5·23	4·98	4·77, 5·19
% Carbohydrates	42·2	40·9, 43·5	44·1	42·5, 45·6	44·5*	43·0, 46·0	44·8	43·2, 46·3
Total dietary fibres (g)	27·2	24·8, 29·5	32·8	30·0, 35·7	25·6	23·9, 27·2	28·1	26·0, 30·2
% Proteins	18·3	17·7, 19·0	18·4	17·6, 19·1	17·8	17·0, 18·5	17·9	17·1, 18·7
% Lipids	36·8	35·6, 38·0	34·6	33·2, 36·0	35·1*	33·9, 36·2	34·6	33·1, 36·1
% MUFA	15·5	14·8, 16·2	15·1	14·3, 15·9	14·6†	13·9, 15·3	15·1	14·1, 16·0
% PUFA	6·2	5·9, 6·6	6·8	6·4, 7·2	5·9	5·5, 6·2	6·3	5·9, 6·7
% SFA	12·2	11·5, 12·9	10·1	9·4, 10·7	11·8	11·4, 12·2	10·5	10·0, 11·1
% *Trans*-fatty acids	1·4	1·3, 1·5	1·1	1·0, 1·2	1·3*	1·2, 1·3	1·1	1·0, 1·2
% Alcohol	2·7	2·1, 3·3	2·9	2·2, 3·6	2·7	2·1, 3·2	2·7	2·1, 3·3
Medscore and food groups								
Medscore (arbitrary units)	22·7	21·6, 23·8	27·6	26·4, 28·9	24·1†	23·1, 25·0	27·2	25·8, 28·6
Olives (portions/d)	1·1	0·7, 1·4	1·5	1·2, 1·8	1·0	0·7, 1·2	1·6	1·1, 2·1
Rapeseed oil (portions/d)	0·2	0·1, 0·3	0·2	0·1, 0·3	0·2	0·1, 0·2	0·2	0·1, 0·3
Vegetables (portions/d)	3·8	3·4, 4·3	4·3	3·8, 4·9	4·3	3·9, 4·6	4·3	3·9, 4·8
Vegetable juices (portions/d)	0·3	0·2, 0·4	0·3	0·2, 0·4	0·3	0·2, 0·4	0·2	0·1, 0·4
Legumes, nuts and seeds (portions/d)	1·3	0·9, 1·6	1·9	1·6, 2·3	0·8*	0·7, 1·0	1·2	1·0, 1·4
Whole-grain products (portions/d)	3·2	2·8, 3·6	3·7	3·3, 4·1	2·3*	1·9, 2·8	2·9	2·5, 3·2
Refined-grain products (portions/d)	3·3	2·8, 3·7	1·9	1·6, 2·2	2·7†	2·3, 3·1	1·9	1·6, 2·3
Milk and dairy products (portions/d)	3·1	2·6, 3·6	2·8	2·3, 3·3	2·6†	2·3, 2·9	2·4	2·1, 2·7
Fruits (portions/d)	1·7	1·4, 2·1	2·6	2·1, 3·2	2·5*	2·1, 2·8	2·6	2·2, 3·0
Fruit juices (portions/d)	1·5	1·1, 1·8	1·3	1·0, 1·6	1·1†	0·8, 1·4	1·0	0·7, 1·4
Poultry (portions/d)	0·9	0·7, 1·1	0·8	0·6, 0·9	0·7*	0·6, 0·8	0·6	0·5, 0·7
Fish and seafood (portions/d)	0·6	0·5, 0·8	1·1	0·9, 1·3	0·5	0·4, 0·7	0·9	0·7, 1·0
Red meat/processed meat (portions/d)	1·7	1·4, 1·9	0·8	0·7, 0·9	1·1*	1·0, 1·3	0·7	0·6, 0·8
Eggs (portions/d)	0·5	0·4, 0·5	0·5	0·4, 0·6	0·3*	0·3, 0·4	0·3	0·2, 0·4
Sweets (portions/d)	2·0	0·8, 3·2	1·0	0·6, 1·5	1·3	1·0, 1·7	0·9	0·7, 1·2

* Mean value was significantly different from that for men at baseline (*P* ≤ 0·05; Student's *t* test).

† Mean value was marginally significantly different from that for men at baseline (*P* ≤ 0·10; Student's *t* test).

‡ Including energy-containing foods and drinks.

**Table 3.** Changes in dietary intakes, Mediterranean score (Medscore) and food group intakes in response to the 12-week nutritional intervention programme

	Men (*n* 57)			Women (*n* 51)			Men *v.* women differences		Men *v.* women differences adjusted for baseline value	
Variables	Δ 0–12 weeks	% Change from baseline	*P*	Δ 0–12 weeks	% Change from baseline	*P*	Mean	95 % CI	Mean	95 % CI
Dietary intakes										
Energy intake (kJ)	−1151	−9·0	0·004	−607	−5·9	0·14	−544	−1660, 573	821	−123, 1765
Energy density (kJ/g)*‡	−0·54	−9·8	< 0·0001	−0·08	−1·6	0·58	−0·1†	−0·2, −0·02	−0·03	−0·1, 0·04
% Carbohydrates	2·2	5·2	0·008	−0·05	−0·1	0·96	2·2	−0·1, 4·6	0·5	−1·6, 2·6
Total dietary fibres (g)	5·9	21·7	< 0·0001	2·6	10·2	0·02	3·4†	0·4, 6·3	3·8	0·9, 6·6
% Proteins	0·06	0·3	0·88	0·2	1·1	0·63	−0·1	−1·3, 1·0	0·2	−0·8, 1·2
% Lipids§	−2·5	−6·8	0·001	−0·3	−0·9	0·72	−2·2†	−4·4, −0·02	−0·8	−2·8, 1·1
% MUFA	−0·5	−3·2	0·24	0·5	3·4	0·36	−1·0	−2·4, 0·4	−0·3	−1·5, 0·9
% PUFA	0·5	8·1	0·04	0·5	8·5	0·04	−0·04	−0·7, 0·7	0·3	−0·2, 0·9
% SFA	−2·2	−18·0	< 0·0001	−1·2	−10·2	0·0002	−1·0†	−1·9, −0·2	−0·8	−1·5, −0·05
% *Trans*-fatty acids*	−0·3	−21·4	< 0·0001	−0·1	−7·7	0·03	−0·2†	−0·4, −0·02	−0·09	−0·2, 0·07
% Alcohol	0·3	11·1	0·33	0·1	3·7	0·61	0·1	−0·6, 0·9	0·1	−0·6, 0·8
Medscore and food groups										
Medscore (arbitrary units)	4·9	21·6	< 0·0001	3·2	13·3	< 0·0001	1·7	−0·3, 3·7	0·8	−0·9, 2·6
Olives (portions/d)	0·4	36·4	0·08	0·6	60·0	0·02	−0·2	−0·8, 0·5	−0·1	−0·6, 0·4
Rapeseed oil (portions/d)*§	0·02	10·0	0·75	0·04	20·0	0·54	−0·02	−0·2, 0·1	0·006	−0·1, 0·1
Vegetables (portions/d)	0·6	15·8	0·02	0·06	1·4	0·85	0·6	−0·2, 1·4	0·2	−0·4, 0·9
Vegetable juices (portions/d)	0·02	6·7	0·70	−0·02	−6·7	0·78	0·04	−0·1, 0·2	0·05	−0·1, 0·2
Legumes, nuts and seeds (portions/d)	0·6	46·2	0·0001	0·4	50·0	0·03	0·3	−0·2, 0·7	0·6	0·2, 1·0
Whole-grain products (portions/d)	0·5	15·6	0·03	0·5	21·7	0·02	−0·06	−0·6, 0·5	0·5	0·01, 1·0
Refined-grain products (portions/d)*§	−1·3	−39·4	< 0·0001	−0·8	−29·6	0·0009	−0·5	−1·1, 0·2	−0·2	−0·6, 0·3
Milk and dairy products (portions/d)	−0·2	−6·5	0·39	−0·1	−3·8	0·54	−0·05	−0·7, 0·6	0·2	−0·3, 0·7
Fruits (portions/d)	1·0	58·8	0·0001	0·09	3·6	0·74	0·9†	0·2, 1·6	0·4	−0·3, 1·1
Fruit juices (portions/d)	−0·2	−13·3	0·29	−0·1	−9·1	0·50	−0·05	−0·5, 0·4	0·08	−0·3, 0·5
Poultry (portions/d)	−0·08	−8·9	0·27	−0·1	−14·5	0·20	0·02	−0·2, 0·2	0·1	−0·04, 0·3
Fish and seafood (portions/d)	0·4	66·7	< 0·0001	0·3	60·0	0·0004	0·1	−0·2, 0·3	0·1	−0·1, 0·4
Red meat/processed meat (portions/d)	−0·9	−52·9	< 0·0001	−0·5	−45·5	< 0·0001	−0·4†	−0·7, −0·1	−0·03	−0·2, 0·2
Eggs (portions/d)	−0·004	−0·8	0·93	−0·03	−10·0	0·52	0·03	−0·1, 0·2	0·1	−0·01, 0·2
Sweets (portions/d)*§	−1·0	−50·0	0·05	−0·4	−30·8	0·46	−0·6	−2·1, 0·9	−0·03	−0·6, 0·5

Δ 0–12 weeks, Change following the 12-week nutritional intervention programme.

* Significant interaction between gender and baseline value (*P* ≤ 0·05).

† Significant differences in men *v.* women between 0–12 weeks without adjustment for the baseline value (*P* ≤ 0·05).

‡ Including energy-containing foods and drinks.

§ Analysis was performed on transformed values.

As for the Medscore, both men and women showed increases in response to the intervention but without significant differences among them. With regards to Medscore components, significant differences were observed between men and women for red and processed meat and fruit intakes. The decrease in red and processed meat and the increase in fruit consumption were more pronounced in men than in women. In addition, intakes of legumes, nuts and seeds, whole-grain products and fish and seafood increased while the intake of refined-grain products decreased in both men and women without significant differences between them. Moreover, a significant increase in vegetable intake was only observed in men whereas a significant increase in olive oil and olive intake was only observed in women, in response to the intervention. After statistical adjustment for the baseline value of the response variable, differences observed between men and women for changes in red and processed meat and fruit intakes were no longer significant. Moreover, significant differences were observed between men and women for legumes, nuts and seeds and whole-grain products intakes once adjusted for the baseline value, with greater increases observed for these variables in men than in women.

At the end of the nutritional intervention, the perceived level of adherence to the Mediterranean diet, as determined by visual analogue scale, was not different between men and women (99·8 (sd 24·2) mm in men and 100·1 (sd 25·3) mm in women; *t* = 0·07; *P* = 0·94). A significant and positive association in both men (*r* 0·33; *P* = 0·01) and women (*r* 0·28; *P* = 0·05) was observed between perceived level of adherence to the Mediterranean diet and the actual Medscore calculated after the 12-week nutritional intervention.

Table 4 presents anthropometric and metabolic values at baseline and at the end of the intervention, and Table 5 presents changes in anthropometric and metabolic variables in response to the 12-week nutritional intervention programme, in men and women separately. As shown in Table 5, no significant differences were observed between men and women for anthropometric changes, in response to the nutritional intervention. However, significant decreases were observed for BMI and waist circumference in both men and women. Also, despite the trend for women to decrease their body weight, only men significantly decreased their body weight and percentage of body fat in response to the nutritional intervention. As for metabolic changes, significant differences were found between men and women for total-C:HDL-C and TAG:HDL-C ratios, with greater decreases observed for these variables in men than in women. In addition, results showed significant changes in HDL-C (increase) and in TAG levels and diastolic blood pressure (decreases) in response to the intervention, but only in men. Moreover, differences observed between men and women in total-C:HDL-C and TAG:HDL-C ratios became non-significant after adjustment for the baseline value.

**Table 4.** Anthropometric and metabolic variables at baseline (time = 0) and after the 12-week nutritional intervention programme (time = 12)* (Mean values and 95 % confidence intervals)

	Men				Women			
	Time = 0 (*n* 64)		Time = 12 (*n* 57)		Time = 0 (*n* 59)		Time = 12 (*n* 51)	
Variables	Mean	95 % CI	Mean	95 % CI	Mean	95 % CI	Mean	95 % CI
Body weight (kg)	96·6	92·7, 100·5	95·2	91·4, 99·0	77·9	73·5, 82·2	77·2	72·4, 82·0
BMI (kg/m^2^)	30·8	29·7, 31·9	30·2	29·2, 31·3	29·6	28·0, 31·2	29·4	27·7, 31·1
Body fat (%)†	26·7	25·4, 27·9	25·6	24·2, 26·9	39·2	37·4, 41·0	39·2	37·1, 41·2
Waist circumference (cm)	106·1	103·6, 108·7	104·2	101·7, 106·7	95·8	92·9, 98·8	94·5	91·0, 98·1
HDL-C (mmol/l)‡	1·1	1·1, 1·2	1·2	1·1, 1·3	1·4	1·4, 1·5	1·4	1·4, 1·5
LDL-C (mmol/l)‡	3·6	3·5, 3·8	3·7	3·5, 3·9	3·6	3·5, 3·8	3·6	3·4, 3·8
ApoB (g/l)‡	1·2	1·1, 1·2	1·2	1·1, 1·2	1·1	1·1, 1·2	1·1	1·1, 1·2
Total-C:HDL-C ratio‡	5·1	4·8, 5·3	4·9	4·6, 5·1	4·2	3·9, 4·4	4·1	3·8, 4·3
TAG (mmol/l)‡	1·9	1·7, 2·1	1·6	1·4, 1·8	1·5	1·3, 1·6	1·4	1·2, 1·5
TAG:HDL-C ratio‡	1·8	1·5, 2·0	1·4	1·2, 1·6	1·1	1·0, 1·2	1·0	0·9, 1·1
Systolic blood pressure (mmHg)‡	119·8	116·1, 123·4	119·3	116·5, 122·1	109·2	106·3, 112·1	109·5	106·2, 112·8
Diastolic blood pressure (mmHg)‡	75·6	73·2, 78·0	72·4	70·2, 74·7	70·8	68·7, 72·8	68·9	66·6, 71·2
Fasting glucose (mmol/l)‡	5·3	5·1, 5·4	5·3	5·2, 5·4	5·2	5·0, 5·3	5·2	5·0, 5·4
Fasting insulin (pmol/l)‡	100·5	89·1, 111·8	98·9	87·2, 110·7	88·5	76·6, 100·5	88·2	76·9, 99·5

HDL-C, HDL-cholesterol; LDL-C, LDL-cholesterol; total-C, total cholesterol.

* All anthropometric and metabolic variables were significantly different between men and women at baseline (time = 0), except for LDL-C, apoB, systolic and diastolic blood pressure, fasting glucose and insulin (*P* ≤ 0·05).

† Men (*n* 52) and women (*n* 48) because of missing values.

‡ Metabolic variables: men (*n* 63) and women (*n* 58) because of missing values.

**Table 5.** Changes in anthropometric and metabolic variables in response to the 12-week nutritional intervention programme

	Men (*n* 57)			Women (*n* 51)			Men *v.* women differences		Men *v.* women differences adjusted for baseline value	
Variables	Δ 0–12 weeks	% Change from baseline	*P*	Δ 0–12 weeks	% Change from baseline	*P*	Mean	95 % CI	Mean	95 % CI
Body weight (kg)	−1·0	−1·0	0·0003	−0·6	−0·8	0·06	−0·5	−1·3, 0·3	−0·6	−1·6, 0·3
BMI (kg/m^2^)	−0·3	−1·0	0·0004	−0·2	−0·7	0·03	−0·1	−0·4, 0·1	−0·1	−0·4, 0·1
Body fat (%)‡	−0·7	−2·6	0·003	−0·2	−0·5	0·31	−0·4	−1·0, 0·2	−0·4	−1·4, 0·6
Waist circumference (cm)	−1·3	−1·2	0·01	−1·1	−1·1	0·04	−0·2	−1·7, 1·3	0·02	−1·7, 1·7
HDL-C (mmol/l)	0·05	4·5	0·005	0·01	0·7	0·42	0·04	−0·01, 0·08	0·007	−0·05, 0·06
LDL-C (mmol/l)	−0·03	−0·8	0·62	−0·06	−1·7	0·43	0·02	−0·2, 0·2	0·05	−0·1, 0·2
ApoB (g/l)	−0·02	−1·7	0·33	−0·01	−0·9	0·58	−0·007	−0·05, 0·04	0·002	−0·04, 0·05
Total-C:HDL-C ratio	−0·3	−5·9	< 0·0001	−0·1	−2·4	0·13	−0·2†	−0·4, −0·03	−0·08	−0·3, 0·1
TAG (mmol/l)*§	−0·3	−15·8	0·0002	−0·09	−6·0	0·20	−0·2	−0·4, 0·03	0·01	−0·2, 0·2
TAG:HDL-C ratio*§	−0·3	−16·7	< 0·0001	−0·08	−7·3	0·26	−0·2†	−0·4, −0·04	0·03	−0·1, 0·2
Systolic blood pressure (mmHg)	1·0	0·8	0·41	0·4	0·4	0·74	0·6	−3·0, 4·2	3·9	0·5, 7·4
Diastolic blood pressure (mmHg)	−2·4	−3·2	0·01	−1·6	−2·3	0·10	−0·8	−3·3, 1·8	0·6	−1·9, 3·1
Fasting glucose (mmol/l)*§	0·04	0·8	0·56	0·04	0·8	0·50	−0·007	−0·2, 0·2	0·02	−0·1, 0·2
Fasting insulin (pmol/l)	−1·6	−1·6	0·61	0·4	0·5	0·91	−2·0	−11·2, 7·1	0·3	−8·3, 8·8

Δ 0–12 weeks, Change following the 12-week nutritional intervention programme; HDL-C, HDL-cholesterol; LDL-C, LDL-cholesterol; total-C, total cholesterol.

* Significant interaction between gender and baseline value (*P* ≤ 0·05).

† Significant differences in men *v.* women between 0–12 weeks without adjustment for the baseline value (*P* ≤ 0·05).

‡ Men (*n* 52) and women (*n* 48) because of missing values.

§ Analysis was performed on transformed values.

## Discussion

The aim of the present study was to determine differences between men and women in dietary, anthropometric and metabolic changes, in response to a 12-week nutritional intervention programme promoting the adoption of the Mediterranean diet, and based on the SDT. Results showed that our nutritional intervention led to improvements in dietary, anthropometric and metabolic profile, that were generally more pronounced in men than in women.

The present results indicate that both men and women increased their level of adherence to the Mediterranean diet (Medscore) in response to the 12-week nutritional intervention and therefore both men and women improved the general quality of their diet. This improvement in the level of adherence to the Mediterranean diet also indicates that our nutritional intervention programme based on the SDT appears to be appropriate for both men and women. Moreover, the significant association found in both men and women between perceived level of adherence to the Mediterranean diet and the actual Medscore calculated suggests that men and women had a similar understanding of the intervention and also the capability to assess the quality of their diet accurately after the end of the 12-week nutritional intervention, which represents relevant information in a context of nutritional education.

Although men and women improved their dietary intakes as shown by the increase in the Medscore, differences were observed between men and women when examining individual components of the Medscore, which were concordant with changes observed in nutritional intakes in response to the intervention. Indeed, the more pronounced changes observed in men in some food groups, for example, by greater decrease in red and processed meat and greater increase in fruit consumption, were consistent with some differences observed between men and women in nutrient intakes such as the greater decreases in energy density, percentage of energy provided by lipids, SFA and *trans*-fatty acids, and the greater increase in fibre intake in men than in women. Moreover, the significant decrease in energy density in men was concordant with the decrease in daily energy intake, as previous studies have reported that decreasing energy density of the diet leads to a spontaneous decrease in energy intake^(^^27^^)^. Similarly to our findings, results from a nutritional intervention, which promoted the traditional Mediterranean diet over a period of 12 months among a Spanish population, reported a greater success in men than in women when considering the level of adherence to the Mediterranean diet^(^^12^^)^. On the other hand, the present results are different from other studies^(^^13^^,^^28^^)^ reporting greater dietary changes in women than in men. Several differences in the intervention design can explain that differences observed between men and women regarding changes in dietary intakes in response to our nutritional intervention programme differ, for example, from the study of Bemelmans *et al.*^(^^13^^)^. First, our intervention included group but also individual counselling sessions to individualise dietary objectives and strategies adopted and to support men and women to overcome barriers in the adoption of the Mediterranean diet, whereas only group sessions were provided to men and women in the study of Bemelmans *et al.*^(^^13^^)^. Second, our nutritional intervention aimed at promoting autonomy and competence in men and women towards the adoption of the Mediterranean diet. Indeed, we did so by supporting them in their dietary changes and strategies to achieve these changes and by promoting the development of their skills and knowledge related to nutrition, which contrasts with specific nutritional guidelines and daily intake explained at the beginning of the intervention in the study of Bemelmans *et al.*^(^^13^^)^. It can be argued that providing more details about dietary guidelines may reduce the possibility for autonomy and that a more passive role of subjects in a nutritional intervention could explain divergence in results obtained among studies. Globally, direct comparison of the present results with those from the literature remains difficult because of major differences in the intervention design and statistical analyses among studies.

Some baseline characteristics in men and women may have influenced the magnitude of dietary changes observed in response to our nutritional intervention. Accordingly, healthier diet at baseline of a dietary intervention has been previously reported to decrease the likelihood of observing significant dietary changes^(^^12^^)^. The fact that women in the present study had globally dietary intakes of higher quality at baseline and which tended to be closer to the traditional Mediterranean diet pattern than those of men could thus possibly explain, at least partially, differences observed between men and women in dietary changes. In the context of our nutritional intervention, it can be hypothesised that because men's dietary intakes at baseline were generally further away from Mediterranean diet principles, they could possibly identify more easily changes that could be made and modify their eating habits, especially in the context of individual counselling sessions where specific dietary objectives were settled. Accordingly, the fact that many differences observed between men and women were no longer significant once dietary changes were adjusted for the baseline value of the response variable brings support to this hypothesis. The present results underline the importance of considering the dietary profile of men and women before the beginning of a nutritional intervention to properly respond to the clients' needs and maximise potential improvements in dietary intakes during the intervention. However, because some differences between men and women in dietary changes were significant once adjusted for the baseline value of the response variable, it is suggested that differences between men and women regarding other factors than baseline dietary intakes, such as attitudes and beliefs towards health and nutrition, might have influenced their response to the nutritional intervention. Our hypothesis is in agreement with a previous study^(^^29^^)^ reporting that health attitudes and beliefs are relevant predictors of adherence to health recommendations. The use of mixed methods in which qualitative and quantitative data are combined would therefore warrant to be considered in the future to obtain more specific information about such factors in men and women.

Although our nutritional intervention promoted healthy dietary changes with no focus on body weight, both men and women showed improvements in their anthropometric profile with significant decreases in BMI and waist circumference, in response to the 12-week nutritional intervention. The present results are concordant with previous studies, which found that a higher adherence to the Mediterranean diet was associated with lower prevalence of overweight or obesity^(^^30^^,^^31^^)^. The Mediterranean diet is recognised to be highly satiating^(^^31^^,^^32^^)^ and the present results suggest that dietary changes led to increased satiety. More specifically, increases in intakes of legumes, nuts and seeds and whole-grain products reported in both men and women possibly contributed to a decrease in energy density through increased water content and fibre intake^(^^33^^)^. Moreover, the decrease in red and processed meat intake may have led to replacement of animal proteins by vegetable proteins sources such as legumes, nuts and seeds, which contain satiating components such as proteins and fibres^(^^34^^,^^35^^)^.

As for metabolic profile, the present results showed more pronounced changes in metabolic variables in men than in women and these can possibly be explained by greater dietary changes observed in men, in response to the nutritional intervention. As suggested by Estruch *et al.*^(^^1^^)^, potential synergy among nutrient-rich foods included in the Mediterranean diet might foster favourable changes in some pathways of cardiovascular risks. It is also possible that some sex-related characteristics such as the level of sex hormones may interact with the complex synergistic effect between food components, resulting in a smaller beneficial impact of the Mediterranean diet in women than in men. In support of this, we recently reported significant improvements in insulin homeostasis in men only, in response to an isoenergetic controlled experimental diet based on the traditional Mediterranean diet where all foods and drinks were provided to the participants^(^^7^^)^. However, the absence of differences between men and women in metabolic changes once statistical adjustment was performed for the baseline value of the response variable underlines the importance of the metabolic status at the beginning of a nutritional intervention programme. Indeed, results related to metabolic changes suggest that an individual with more deteriorated metabolic variables before the beginning of a nutritional intervention could show greater health improvements in response to the intervention, which is concordant with the fact than men in the present study had a more deteriorated metabolic profile at baseline and improved more in response to the intervention than women.

We acknowledge that the present results cannot be extrapolated to the whole population because we recruited men and women presenting risk factors for CVD and who had dietary intakes closer to recommendations of the Canada's Food Guide recommendations than the general adult population in Canada^(^^36^^)^. Moreover, although anthropometric and metabolic variables were measured, dietary intakes were self-reported. Therefore, the risk of misreporting dietary intakes cannot be excluded. The present study has also important strengths such as the fact that analyses were conducted distinctively in men and women according to dietary intakes, anthropometric variables and metabolic profile in the context of a nutritional intervention programme. Also, our nutritional intervention based on the SDT appears to be acceptable for both men and women as similar attrition and attendance rate to the intervention as well as to its specific components (i.e. group meetings, individual sessions, follow-up telephone calls) was observed among them. Although attrition rate found in the present study was similar to those reported in the literature among nutritional interventions based on a motivational interviewing approach^(^^37^^)^, reasons for not attending some sessions remain difficult to identify and might differ between men and women. It remains essential to consider the clinical implication related to attendance rate when developing a nutritional intervention. Indeed, the lack of flexibility in the schedule related to group meeting attendance must be considered in the development of interventions as it may require efforts for some individuals to attend pre-scheduled meetings and this might progressively generate fatigue with time. It is also possible that active participation in practical activities, for example the cooking lesson, may be perceived as requiring too much effort for some individuals. Nevertheless, the present study underlines the potential of improvement in adherence to the Mediterranean diet among a non-Mediterranean population, more specifically in the context of an intervention during which men and women chose their own dietary objectives (i.e. based on their personal interest and motivations). In addition, the present results bring information about differences between men and women in potential health benefits obtained following a 12-week nutritional intervention programme promoting the Mediterranean diet, and thus support the relevance to consider gender in the development of nutritional intervention programmes aimed at preventing chronic diseases.

### Conclusions

In conclusion, the present results suggest that the nutritional intervention programme promoting the adoption of the Mediterranean diet and based on the SDT led to greater improvements in dietary intakes in men than in women, which appear to have contributed to beneficial anthropometric and metabolic changes, more particularly in men. However, the present results also suggest that the more deteriorated metabolic profile found in men at baseline appears to explain to a large extent the fact that the improvements in CVD risk factors were more pronounced in men than in women in response to the intervention.
